# Cognitive reappraisal and empathy chain-mediate the association between relative deprivation and prosocial behavior in adolescents

**DOI:** 10.3389/fpsyg.2023.1238308

**Published:** 2023-09-22

**Authors:** Yanfeng Xu, Sishi Chen, Xiaojie Su, Delin Yu

**Affiliations:** ^1^School of Psychology, Fujian Normal University, Fuzhou, Fujian, China; ^2^Normal College, Urumqi Vocational University, Urumqi, Xinjiang, China

**Keywords:** relative deprivation, cognitive reappraisal, expressive suppression, empathy, prosocial behavior

## Abstract

**Background:**

Relative deprivation is one of the factors that influences the development of personality and behavior. However, it is still unclear whether and how relative deprivation decreases the prosocial behavior in adolescents. This study aimed to examine the association between relative deprivation and adolescent prosocial behavior and the role of emotion regulation strategies and empathy in modifying this association.

**Methods:**

The present study included 609 secondary school students (*M* = 15.42 years, *SD* = 0.653) in Fujian Province, China. All participants completed the Relative Deprivation Questionnaire, Emotion Regulation Scale, the Basic Empathy Scale, and Prosocial Behavior Scale. The collected data were analyzed using SPSS 25.0 and Mplus 7.4.

**Results:**

Relative deprivation was negatively correlated with cognitive reappraisal, but positively correlated with expressive suppression. Cognitive reappraisal was positively correlated with empathy and prosocial behavior, but expressive suppression was not. Empathy was positively correlated with prosocial behavior. Relative deprivation decreased prosocial behavior through (a) cognitive reappraisal, (b) empathy, and (c) chain mediation of cognitive reappraisal and empathy. No significant mediating effect of expressive suppression was found.

**Conclusion:**

The results indicate that relative deprivation decreases adolescent prosocial behavior, and that cognitive reappraisal and empathy are the potential psychological mechanisms that affect the association between relative deprivation and adolescent prosocial behavior.

## 1. Introduction

Prosocial behaviors are behaviors that are beneficial to others, society, and the nation (Pfattheicher et al., 2022). At the individual level, while one’s prosocial behavior is beneficial to others in difficult situations, it also has potential benefits for oneself, such as increased probability of being assisted (El Mallah, 2020), good community reputation (Berman and Silver, 2022), and a sense of meaning and value in life (Klein, 2017); at the societal level, prosocial behavior contributes to a well-functioning and harmonious society (Carlo and Pierotti, 2020). Given the importance of prosocial behavior, it is considered an essential aspect of social and moral development during adolescence (Hart and Carlo, 2005). Adolescent development is often regarded as a period of social sensitivities that shapes the behavior and character traits of individuals. Therefore, it is necessary to understand the factors that influence the development of adolescent prosocial behavior.

With increased social and economic instability, widening economic disparities, and the rapid growth of online social media and networks, people today may be more likely than ever to make social comparisons and experience feelings of relative deprivation (Power et al., 2020). Relative deprivation refers to the perception of being worse off compared to a certain standard, accompanied by feelings of anger and resentment (Smith et al., 2012). While previous studies have identified that relative deprivation as a significant factor reduces prosocial behavior (Zhang et al., 2016; Pak and Babiarz, 2023; Zhang et al., 2023), but most of the studies mainly focused on adults; there is a lack of research on the adolescents, and the mechanisms underlying the association between relative deprivation and prosocial behavior is still unclear. Adolescence is a special stage of rapid development of self-identity and self-awareness. It is also the period when individuals are susceptible to developing a sense of relative deprivation because of social comparison (Orben et al., 2020). Exploring the pathways through which relative deprivation affects adolescent prosocial behavior would help school mental health teachers implement effective interventions. Thus, the main question addressed in the present study is if and how relative deprivation decreases the prosocial behavior among adolescents.

## 2. Literature review

### 2.1. Relative deprivation and prosocial behavior

Previous research has shown that individuals with relative deprivation often tend to reject prosocial behavior (Pak and Babiarz, 2023). For example, when individuals experience relative deprivation caused by unfair social distribution, they show reduced generosity in dictatorial games (Gheorghiu et al., 2021), and the priming of one’s relative deprivation reduced the meaningfulness of engaging in prosocial behavior (Zhang et al., 2023). This may be due to relative deprivation highlights individuals’ self-perception as victims of injustice and directs their attention to perceived disadvantages (Callan et al., 2017). According to the social information processing model, before engaging in prosocial behavior, individuals first assess their situation, for instance, whether their needs are being met and whether they have sufficient capacity to engage. The preliminary judgment would determine whether they ultimately engage in prosocial behavior (Nelson and Crick, 1999). Individuals suffering relative deprivation, usually develop a cynical view of society and become more self-centered and less concerned about the plight of others (Zitek et al., 2010). Zhang et al. (2016) validated this opinion, suggesting that the tendency to prioritize self-interest over others mediated the effect of relative deprivation on prosocial behavior. Based on the consistent findings from previous studies involving adults, the present study proposes hypothesis H1: Relative deprivation negatively predicts adolescent prosocial behavior.

### 2.2. Emotion regulation strategies as a mediator

Relative deprivation results in anxiety and attention bias toward a threat (Zhang et al., 2021), which may indicate that individuals with high levels of relative deprivation are more inclined to engage in automatic negative thinking and develop avoidance attitudes when solving emotional problems (Nadler et al., 2020). The theory of emotion suppression proposed by Langner et al. (2012) may explain this phenomenon. When individuals with low social status subjectively perceive themselves to be in a worse position than others, they usually hide or suppress their negative feelings and behaviors to avoid showing their dissatisfaction and anger in the presence of others with high status. Consistently, Liu et al. (2021) also found that individuals with high levels of relative deprivation tend to use expressive suppression strategies to moderate the emergence of negative emotions in more situations, and rarely use cognitive reappraisal strategies.

The use of emotion regulation strategies could predict prosocial behavior. Previous research has revealed that maladaptive emotion regulation strategies (e.g., expressive suppression) are negatively associated with prosocial behavior (Lockwood et al., 2014), whereas the use of adaptive emotion regulation (e.g., cognitive reappraisal) is positively associated with prosocial behavior (Hodge et al., 2023). This phenomenon also occurs in adolescents; Li et al. (2021) found that adolescents who applied cognitive reappraisal tended to be more likely to engage in prosocial behavior than those who applied expressive suppression. This finding could be attributed to the fact that compared to adaptive emotion regulation strategies, maladaptive strategies distract the person and obscure important information on social interactions to the detriment of prosocial behavior (Shaver et al., 2008).

Taken together, it could be inferred that the use of emotion regulation strategies plays a crucial role in the association between relative deprivation and prosocial behavior. However, this opinion remains to be confirmed. In the present study, we focus on two widely studied emotion regulation strategies: expressive suppression and cognitive reappraisal. Previous studies have shown that the two emotion regulation strategies operate in contrasting effects (Zhou et al., 2023), and differ in terms of psychological processes and physiological mechanisms (Bebko et al., 2011; Hermann et al., 2014). Considering with these findings and referencing relevant literature (Zhang et al., 2023), the present study explored the mediating effect of cognitive reappraisal and/or expressive suppression, respectively. Therefore, the present study proposes the following hypothesis H2 and H3: Cognitive reappraisal mediates the association between relative deprivation and adolescent prosocial behavior (H2), and expressive suppression mediates the association between relative deprivation and adolescent prosocial behavior (H3).

### 2.3. Empathy as a mediator

Many studies have demonstrated the association between feelings of relative deprivation and counter-empathy (e.g., envy and schadenfreude; Leach and Spears, 2009; Neufeld and Johnson, 2016; Zhao and Zhang, 2022). Relative deprivation arises from perceived inequality and can easily lead to anger and resentment—the core components of malicious envy (Lange and Crusius, 2015). According to the deservingness theory (Feather, 1999), when people become aware of their disadvantaged status, their dissatisfaction with injustice might cause them to feel schadenfreude, especially if they see those in a superior position as undeserving (Feather and Nairn, 2005). Fu et al. (2017) argued that there may be a common psychological mechanism underlying empathy and counter-empathy. Although there is an unproven association between relative deprivation and empathy, based on the demonstrated association between relative deprivation and counter-empathy, it can be theorized that relative deprivation would negatively predict empathy.

The empathy-altruism hypothesis emphasizes that empathy is a direct cause of prosocial behavior (Batson, 2017). Specifically, when individuals feel empathy toward someone in need, they are motivated to take action to help alleviate their suffering, without any expectation of receiving something in return. The results from various types of previous studies have confirmed the substantial association between empathy and prosocial behavior (Davis, 2015). For example, Tang (2015) found that empathy training with primary school students led to a significant increase in the frequency of prosocial behavior. Furthermore, a longitudinal study with adolescents conducted by Wang and Wu (2020) showed that T1 trait empathy competence significantly predicted T2 prosocial behavior. Recently, a meta-analysis including 62 studies revealed an above moderate-strength positive correlation between empathy and prosocial behavior (Yin and Wang, 2023). The evidence from these studies supports the empathy-altruism hypothesis, namely that empathy is an important motivating factor for prosocial behavior.

Combining with the above, adolescents with high levels of relative deprivation may lack the motivation to engage in prosocial behavior owing to low levels of empathy. Hence, the present study proposes hypothesis H4: empathy mediates the association between relative deprivation and adolescent prosocial behavior.

### 2.4. A chain-mediation model

Decety and Michalska (2010) argue that emotion regulation is an important component of the empathy process. The ability of well-regulated individuals to regulate negative emotions and maintain optimal levels of emotional arousal allows them to increase their attention to situations faced by others. A close association between emotion regulation and empathy has been identified in the previous literature, e.g., Ornaghi et al. (2020) confirmed that emotion regulation skills play an important role in promoting empathy development in children. Thompson et al. (2019) proposed an integrative account of empathy and emotion regulation—this theory suggests effective regulation of one’s own emotions can facilitate empathy for others by enabling individuals to better understand and respond to the emotions of others. For instance, if someone could regulate their own feelings of anger well, they may be more capable of empathizing with someone else’s anger and provide more compassionate support. Consistent with this theory, Benita et al. (2017) found the ability to emotion regulation promotes prosocial behavior through the mediation of empathy.

For adolescents, cognitive reappraisal is thought to have a positive emotion-regulation effect (Compas et al., 2017), and, thus, promotes empathy and further increases prosocial behavior. Conversely, expressive suppression is thought to have a negative emotion-regulation effect (Schäfer et al., 2017), and, thus, reduces empathy and further decreases prosocial behavior. Since the use of the two emotion regulation strategies is influenced by one’s perceived relative deprivation, cognitive reappraisal and/or expressive suppression and empathy may play a chain-mediating role in relative deprivation and adolescent prosocial behavior. Therefore, the present study proposes hypotheses H5 and H6: Cognitive reappraisal and empathy have a chain-mediating effect on the association between relative deprivation and prosocial behavior (H5); expressive suppression and empathy have a chain-mediating effect on the association between relative deprivation and prosocial behavior (H6).

### 2.5. Summary

**Figure 1:**
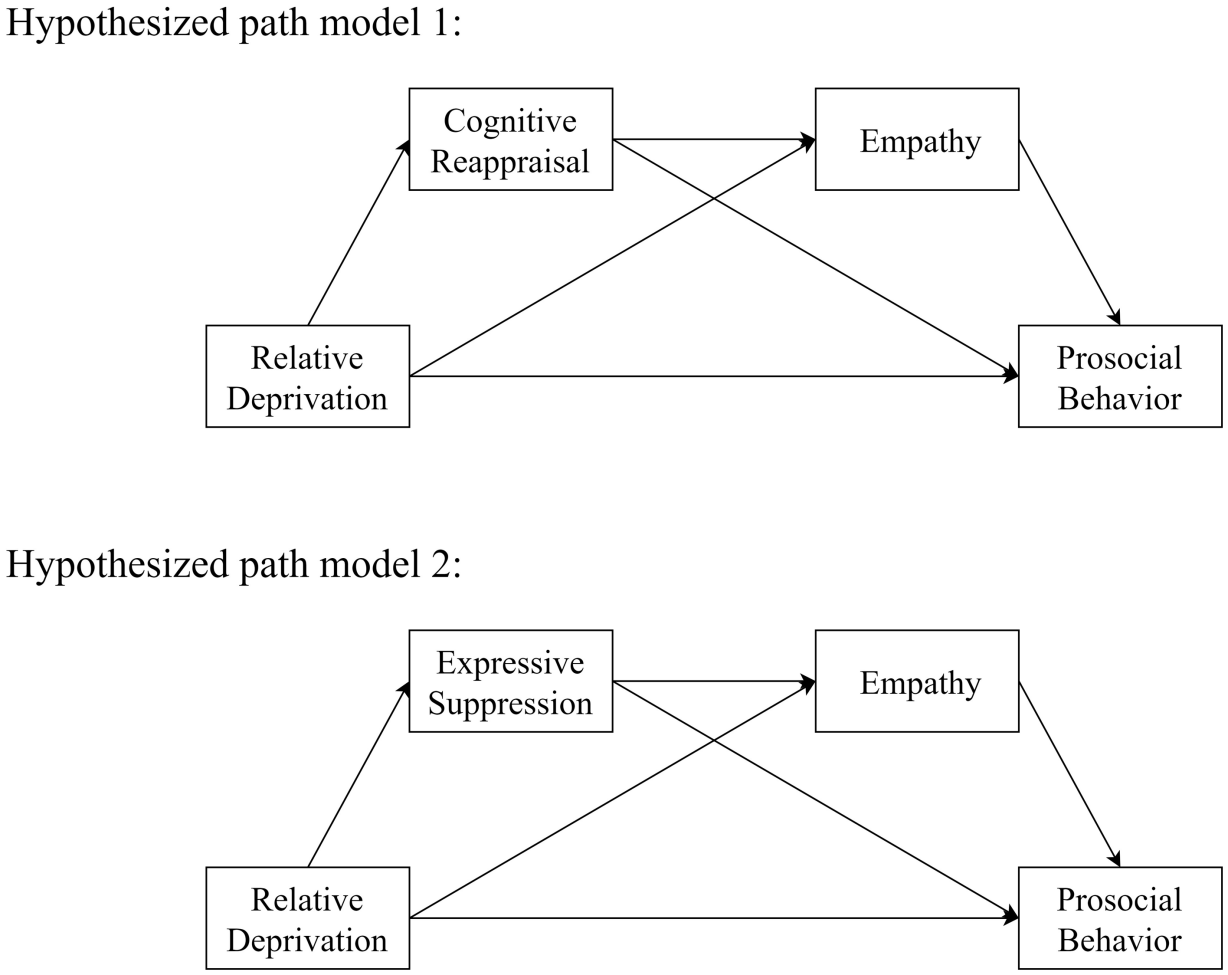
The hypothetical model diagram of this study.

The association between relative deprivation and prosocial behavior in adolescents has not been examined, and the mechanisms underlying this association are unclear. By combining previous theoretical perspectives and empirical evidence, the present study sought to construct two chain mediation models. As shown in Figure 1, the objectives of the study were as follows: (a) to determine whether relative deprivation was a negative predictor of adolescent prosocial behavior; (b) to determine whether emotion regulation strategies and empathy act as chain mediators, with relative deprivation predicting cognitive reappraisal/expressive suppression, which in turn predicts empathy, and ultimately predicts adolescent prosocial behavior.

## 3. Methods

### 3.1. Participants

This study was conducted with high school students in a middle school in Fujian Province, China. Regarding the sample size required for structural equation modeling (SEM), Comrey and Lee (1992) stated that a sample size of 50 is very poor, 100 is poor, 200 is fair, 300 is good, and 500 is very good. The total number of questionnaire items in this study was 51. Therefore, based on the rule of thumb and the “10-times rule” (Hair et al., 2011), the sample size of this study should be greater than 510. Considering the risk of non-return of questionnaire, 650 questionnaires were distributed, and 633 questionnaires were successfully returned, with a return rate of 97.4%. Based on the regularity of questionnaire responses, invalid questionnaires (e.g., the same answer for nearly every question) were excluded, and 609 questionnaires were deemed valid, with an efficiency rate of 93.7%. In the valid samples, the average age was 15.42 years (SD = 0.653). In terms of gender distribution, 47.3% of participants were males and 52.7% were females, which closely resembled the gender ratio of high school students.

### 3.2. Instruments

#### 3.2.1. Relative deprivation questionnaire

As previous studies have confirmed that cultural differences exist in relative deprivation (Smith et al., 2018), we used the Relative Deprivation Questionnaire (RDQ) developed by Ma (2012). The RDQ is based on the general population of China, and its items are aligned with collectivist cultural perceptions of relative deprivation (Van den Bos et al., 2015), e.g., “Most of those rich people in society got rich through dishonorable means.” It has been applied to adolescents in previous studies and demonstrated good psychometric properties (Yang et al., 2021). The RDQ consists of four items in a single dimension, scored using a 6-point scale that ranges from 1 (strongly disagree) to 6 (strongly agree). The higher the total score, the greater the relative deprivation. In the present study, the Cronbach’s alpha coefficient for the RDQ was 0.704, indicating acceptable internal consistency (Taber, 2018).

#### 3.2.2. Emotion regulation scale

We used the Emotion Regulation Scale (ERS), developed by Wang et al. (2007), to measure cognitive reappraisal and/or expressive suppression in adolescents. It is based on the emotion regulation model of Gross (1999) and has been revised to align with Chinese culture. The ERS has good reliability and measurement equivalence for the adolescent population (Chen et al., 2023), and it has been widely used to measure emotion regulation strategies in Chinese adolescents (Wang et al., 2022). The ERS consists of 14 items, including two dimensions (cognitive reappraisal/expressive suppression), with seven questions each. Participants rate their agreement with each statement using a 7-point Likert scale from 1 (strongly disagree) to 7 (strongly agree). Higher scores on each subscale indicate a greater tendency to use the corresponding emotion regulation strategy. In the present study, the Cronbach’s alpha coefficients for the total scale, cognitive reappraisal subscale, and expressive suppression subscale were 0.833, 0.865, and 0.730, respectively, indicating good internal consistency.

#### 3.2.3. Basic empathy scale

The Basic Empathy Scale (BES) was developed by Jolliffe and Farrington (2006) for adolescents. Compared to previous empathy measurement tools, the BES focused on both affect congruence (affective empathy) and understanding of another person’s emotions (cognitive empathy) that more accurately conform to the concept of empathy (Jolliffe and Farrington, 2006). The present study used the Chinese version translated by Li et al. (2011) that has shown good psychometric properties in previous studies with Chinese adolescents (Yu et al., 2020). The BES consists of two dimensions and 20 items, specifically nine items on cognitive empathy and 11 items on affective empathy. Participants rate each item on a 7-point Likert scale, ranging from 1 (strongly disagree) to 7 (strongly agree). Higher scores indicate higher levels empathy. In the present study, the Cronbach’s alpha coefficients for the total scale, cognitive empathy subscale, and affective empathy subscale were 0.818, 0.789, and 0.757, respectively, indicating good internal consistency.

#### 3.2.4. Prosocial behavior scales

Regarding the conceptual structure of the Chinese adolescents’ Prosocial Behavior Scale (PBS), besides the altruism dimension reflecting purely the interest of others, it includes the compliance dimension reflecting adherence to societal norms or organizational rules, the relationship dimension reflecting interpersonal harmony in social interactions, and the personal trait dimension reflecting the motivation for self-improvement. Therefore, the PBS developed by Yang et al. (2016) can accurately measure Chinese adolescent prosocial behavior. It has been widely used to measure prosocial behavior in Chinese adolescents (Zhou et al., 2020). The PBS consists of altruism (four items), compliance (five items), relationship (three items), and personal traits (three items). Participants rate each item on a 7-point Likert scale, ranging from 1 (strongly disagree) to 7 (strongly agree). Higher scale scores indicate increased prosocial behavior. In the present study, the Cronbach’s alpha coefficients for the total scale, altruism subscale, compliance subscale, relationship subscale, and personal trait subscale were 0.907, 0.754, 0.777, 0.631, and 0.724, indicating good internal consistency.

### 3.3. Procedures and statistical analysis

The present study used the cluster random sampling method. Students from 13 classes in the first and the second grades of high school were selected through a random number table. The test was administered by two graduate students majoring in psychology, and the instructions were read out after the questionnaires were distributed; the questionnaires were collected within the specified time.

After data collection, we calculated the descriptive statistics (i.e., mean score and standard deviation) and bivariate correlation of the variables using SPSS 25. The present study used self-report scales to collect the data that may lead to common method bias (Lindell and Whitney, 2001). All items were included in the exploratory factor analysis, according to Harman’s single-factor test for the common method bias. Mplus 7.4 offers powerful analytical capabilities and flexibility in latent variable modeling, which can cope with multivariate, multidimensional, and multilevel analytic needs; it provides comprehensive model fitting and diagnostic information to help researchers gain a deeper understanding and interpretation of latent variable structures and relationships (Wang and Wang, 2019). Therefore, the following statistical analyses involving latent variables were conducted using Mplus 7.4. Referring to the guideline proposed by Rönkkö and Cho (2022), we used confirmatory factor analysis (CFA) to assess discriminant validity. As SEM has the advantages of independent variables containing measurement errors, high precision of parameter estimation, and rich evaluation indexes for model fitting, it was used to examine the mediating effect of cognitive reappraisal/expressive suppression and empathy on the association between relative deprivation and prosocial behavior. The PBS was parceled into four items according to the four subscales, and the BES was parceled into two items according to the two subscales. As suggested by Wu and Wen (2011), the single dimensional RDQ, the cognitive reappraisal subscale, and expressive suppression subscale were each parceled into two items using the odd-even method. The significance of the mediating effect was analyzed by the bootstrap method, with sampling for 5,000 times, and 95% confidence intervals were calculated; if the confidence interval did not include zero, it indicated a significant effect (Cheung, 2007).

## 4. Results

### 4.1. Common method bias and discriminant validity

For common method bias, the unrotated exploratory factor analysis extracted 11 factors with eigenvalues greater than 1. The first factor explained 17.87% of the variance (below the critical threshold of 40%), indicating that no serious common method biases were present in the data. For discriminant validity, as shown in Table 1, all correlations between the factors in the CFA were smaller than the square values of the average variance extracted (AVE) for each factor, thus satisfying the Fornell and Larcker (1981) criterion.

**Table 1:** Descriptive statistics and correlation coefficients for all variables.

Variables	Mean	SD	1	2	3	4	5
1 Relative Deprivation	12.50	4.06	0.762	−0.193^***^	0.235^***^	−0.295^***^	−0.365^***^
2 Cognitive Reappraisal	31.24	7.86	−0.143^***^	0.904	0.423^***^	0.283^***^	0.312^***^
3 Expressive Suppression	27.93	8.26	0.178^***^	0.355^***^	0.800	−0.009	−0.046
4 Empathy	70.10	9.63	−0.149^***^	0.168^***^	−0.075	0.632	0.607^***^
5 Prosocial Behavior	72.18	14.45	−0.287^***^	0.279^***^	−0.031	0.442^***^	0.858

Pearson zero-order correlations for all variables are presented below the diagonal, and factor correlations for all variables are presented above the diagonal. The diagonal is the square value of the Average Variance Extracted (AVE). According to the decision rule presented by Fornell and Larcker (1981), discriminant validity holds for two scales if the square values of the AVE for both are higher than the factor correlation between the scales.

^***^p < 0.001.

### 4.2. Correlation analysis between variables

Pearson zero-order correlations between the variables were calculated (Table 1). Relative deprivation was negatively correlated with cognitive reappraisal (*r* = −0.143, *p* < 0.001), empathy (*r* = −0.149, *p* < 0.001), and prosocial behavior (*r* = −0.287, *p* < 0.001), but positively correlated with expressive suppression (*r* = 0.178, *p* < 0.001). Cognitive reappraisal was positively correlated with expressive suppression (*r* = 0.355, *p* < 0.001), empathy (*r* = 0.168, *p* < 0.001), and prosocial behavior (*r* = 0.279, *p* < 0.001). Expressive suppression was not significantly correlated with empathy (*r* = −0.075, *p* = 0.065) and prosocial behavior (*r* = −0.031, *p* = 0.447). Empathy was positively correlated with prosocial behavior (*r* = 0.442, *p* < 0.001).

### 4.3. Chain-mediating effect of cognitive reappraisal and empathy

**Figure 2:**
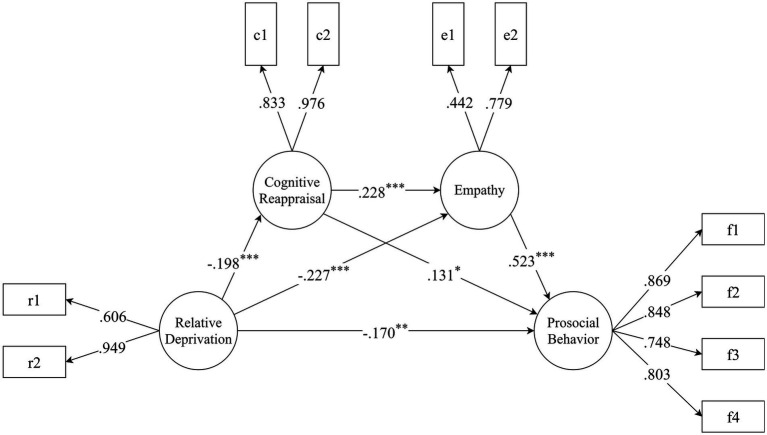
The path diagram of hypothesized model 1 (cognitive reappraisal and empathy as mediators). The structural equation model linking relative deprivation and prosocial behavior through cognitive reappraisal and empathy. The PBS was parceled into four items according to the four subscales, and the BES was parceled into two items according to the two subscales. As suggested by Wu and Wen (2011), the unidimensional RDQ and cognitive reappraisal subscale into two items using the odd-even method. Pathway coefficient and factors loadings are standardized. ^*^p < 0.05, ^**^p < 0.01, ^***^p < 0.001.

The hypothesized path model 1 comprised 10 observed variables and 4 latent variables (relative deprivation, cognitive reappraisal, empathy and prosocial behavior). This model showed an excellent fit with the data: *χ*^2^/*df* = 3.902, *RMSEA* = 0.069, *CFI* = 0.969, *TLI* = 0.952, and *SRMR* = 0.031. The results (Figure 2) showed that (1) relative deprivation negatively predicted cognitive reappraisal (*β* = −0.198, *p* < 0.001), empathy (*β* = −0.227, *p* < 0.001) and prosocial behavior (*β* = −0.170, *p* = 0.003); (2) cognitive reappraisal positively predicted empathy (*β* = 0.228, *p* < 0.001) and prosocial behavior (*β* = 0.131, *p* = 0.016); and (3) empathy positively predicted prosocial behavior (*β* = 0.523, *p* < 0.001). The bootstrapping estimates of 95% confidence intervals (CIs) of the model pathways and mediating effect are shown in Table 2. The mediating effect of cognitive reappraisal was significant (effect = −0.026, *p* = 0.041), accounting for 7.67% of the total effect. The mediating effect of empathy was significant (effect = −0.119, *p* = 0.007), accounting for 35.10% of the total effect. The chain-mediating effect of cognitive reappraisal and empathy was also significant (effect = −0.024, *p* = 0.021), accounting for 7.08% of the total effect.

**Table 2:** Bootstrapping estimates of 95% confidence intervals of (CI) estimation for the model pathways and indirect effect (model 1).

Model pathways	Boot lower 2.5%	Effect	Boot upper 2.5%	*p*-value
Relative deprivation → Prosocial behavior	−0.277	−0.170	−0.057	< 0.001
Relative deprivation → Cognitive reappraisal	−0.306	−0.198	−0.097	< 0.001
Relative deprivation → Empathy	−0.365	−0.227	−0.104	< 0.001
Cognitive reappraisal → Prosocial behavior	0.014	0.131	0.229	0.016
Cognitive reappraisal → Empathy	0.088	0.228	0.361	< 0.001
Empathy → Prosocial behavior	0.361	0.523	0.671	< 0.001
Mediating effect of cognitive reappraisal	−0.058	−0.026	−0.006	0.041
Mediating effect of empathy	−0.224	−0.119	−0.051	0.007
Chain-mediating effect of cognitive reappraisal and empathy	−0.055	−0.024	−0.009	0.021

### 4.4. Chain-mediating effect of expressive suppression and empathy

**Figure 3:**
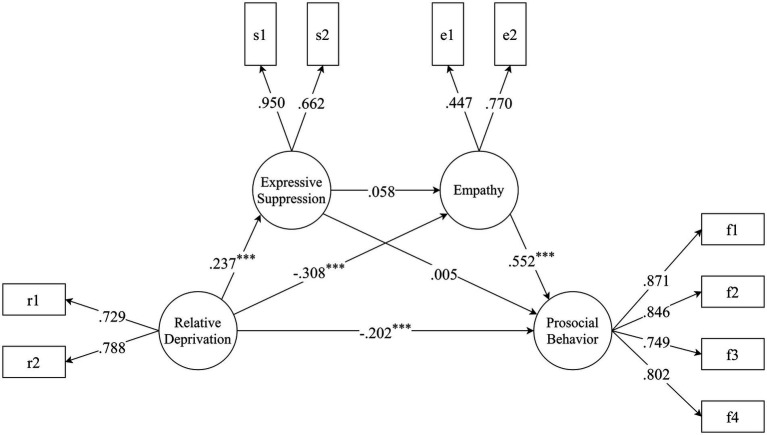
The path diagram of hypothesized model 2 (expressive suppression and empathy as mediators). The structural equation model linking relative deprivation and prosocial behavior through expressive suppression and empathy. The PBS was parceled into four items according to the four subscales, and the BES was parceled into two items according to the two subscales. As suggested by Wu and Wen (2011), the unidimensional RDQ and expressive suppression subscale were each parceled into two items using the odd-even method. Pathway coefficient and factors loadings are standardized. ^*^p < 0.05, ^**^p < 0.01, ^***^p < 0.001.

The hypothesized path model 2 comprised 10 observed variables and 4 latent variables (relative deprivation, expressive suppression, empathy and prosocial behavior). This model showed an acceptable fit with the data: *χ*^2^/*df* = 5.384, *RMSEA* = 0.085, *CFI* = 0.946, *TLI* = 0.916, and *SRMR* = 0.044. The results (Figure 3) showed that (1) relative deprivation positively predicted expressive suppression (*β* = 0.237, *p* = 0.002), but negatively predicted empathy (*β* = −0.308, *p* < 0.001) and prosocial behavior (*β* = −0.202, *p* = 0.002); (2) expressive suppression not significantly predicted empathy (*β* = 0.058, *p* = 0.535) and prosocial behavior (*β* = 0.005, *p* = 0.939); (3) empathy positively predicted prosocial behavior (*β* = 0.552, *p* < 0.001). The bootstrapping estimates of 95% CIs of the model pathways and mediating effect are shown in Table 3. The mediating effect of expressive suppression was not significant (effect = 0.001, *p* = 0.942). The mediating effect of empathy was significant (effect = −0.170, *p* = 0.001), accounting for 46.68% of the total effect. The chain-mediating effect of expressive suppression and empathy was not significant (effect = 0.008, *p* = 0.581).

**Table 3:** Bootstrapping estimates of 95% confidence intervals of (CI) estimation for the model pathways and indirect effect (model 2).

Model pathways	Boot lower 2.5%	Effect	Boot upper 2.5%	*p*-value
Relative deprivation → Prosocial behavior	−0.320	−0.202	−0.068	0.002
Relative deprivation → Expressive suppression	0.064	0.237	0.368	0.002
Relative deprivation → Empathy	−0.453	−0.308	−0.165	< 0.001
Expressive suppression → Prosocial behavior	−0.105	0.005	0.135	0.939
Expressive suppression→ Empathy	−0.149	0.058	0.213	0.535
Empathy → Prosocial behavior	0.361	0.552	0.673	< 0.001
Mediating effect of expressive suppression	−0.026	0.001	0.036	0.942
Mediating effect of empathy	−0.299	−0.170	−0.090	0.001
Chain-mediating effect of expressive suppression and empathy	−0.015	0.008	0.039	0.581

## 5. Discussion

The present study revealed that both cognitive reappraisal and empathy separately mediate the association between relative deprivation and adolescent prosocial behavior. Additionally, cognitive reappraisal and empathy chain-mediate the association between relative deprivation and adolescent prosocial behavior. However, expressive suppression was not found to mediate the association between relative deprivation and adolescent prosocial behavior.

### 5.1. Cognitive reappraisal rather than expressive suppression plays a mediating role

Supporting the hypothesis of this study, we found that relative deprivation significantly predicted cognitive reappraisal negatively, which is consistent with the results of Liu et al. (2021). This finding suggests that individuals with higher relative deprivation are less likely to use cognitive reappraisal when emotions need to be regulated. From the perspective of social comparison theory (Suls and Wheeler, 2012), when adolescents engage in comparisons with their peers, if they perceive themselves to be at a relative disadvantage or facing unfair circumstances, it activates negative thinking patterns and increases the likelihood of adopting negative coping strategies (Xiong et al., 2022).

The results showed that cognitive reappraisal significantly predicted prosocial behavior positively and mediated the association between relative deprivation and prosocial behavior, which is consistent with the hypothesis of this study. Cognitive reappraisal is considered an adaptive strategy that helps individuals perceive situations in a positive light and reduce the impact of negative emotions (Sun et al., 2020). According to the broaden-and-build theory of positive emotions (Fredrickson, 1998), positive emotional states play a role in expanding attention and awareness as well as promoting prosocial behavior. Previous empirical research has also confirmed the link between cognitive reappraisal and prosocial behavior (Li et al., 2021; Hodge et al., 2023). Thus, adolescents with high relative deprivation tend to use cognitive reappraisal strategies less frequently and, resultantly, are less likely to engage in prosocial behavior.

Interestingly, our results revealed that relative deprivation significantly predicted expressive suppression positively, but expressive suppression did not predict prosocial behavior. Moreover, there was no significant mediating effect of expressive suppression on the association between relative deprivation and prosocial behavior, a finding that contradicted the hypothesis of this study. According to the theory of emotion suppression proposed by Langner et al. (2012), when individuals with low social status subjectively believe that they are in a worse position than others, they usually choose to hide and suppress their negative emotions and behavioral expressions (i.e., using the expression suppression strategy) in order to prevent showing their dissatisfaction and anger in front of other individuals with higher status and reduce the risk of conflict between the disadvantaged and dominant groups. This theory explains the relationship between relative deprivation and expressive suppression. However, expressive suppression strategies do not mitigate negative emotional experience and only serve to inhibit the outward manifestations of negative behaviors (Gross and Cassidy, 2019). Thus, the use of expressive suppression strategies may not explain the effect of relative deprivation on prosocial behavior.

### 5.2. Chain mediation role of cognitive reappraisal and empathy

Consistent with the hypothesis of this study, we found that empathy significantly predicts prosocial behavior positively and mediated the association between relative deprivation and prosocial behavior. When adolescents experience relative deprivation, their self-concept is threatened (Kural and Kovács, 2022), which inhibits their empathic concern for others in need (Krol and Bartz, 2022). This lack of empathy may then lead to reduced prosocial behavior. In addition, individuals with high relative deprivation may also experience high levels of counter-empathy, such as envy and schadenfreude (Neufeld and Johnson, 2016), which could further inhibit empathy and decrease motivation for prosocial behavior.

Furthermore, the results showed that cognitive reappraisal and empathy chain-mediated the association between relative deprivation and prosocial behavior significantly. According to the integrative account of empathy and emotion regulation (Thompson et al., 2019), the way in which we understand and respond to others’ emotions may be influenced by emotion regulation. Positive and adaptive emotion regulation strategies can reduce negative emotions during the empathy process and facilitate prosocial behavior (Lockwood et al., 2014). Cognitive reappraisal has been found to promote prosocial behavior by mediating empathy in previous studies (Laghi et al., 2018). In the empathy accuracy task, the use of cognitive reappraisal strategies improved individuals’ accuracy in empathizing with others’ negative emotions (Guo et al., 2023). Therefore, adolescents with high relative deprivation may struggle to empathize with others due to a lack of adaptive emotion regulation strategies that decreases the motivation to engage in prosocial behavior. It is worth noting that the chain-mediating effect was partially mediated the association between relative deprivation and prosocial behavior and accounted for a relatively small proportion of the total effect, suggesting that relative deprivation may also influence prosocial behavior through other mechanisms. A more comprehensive understanding of how relative deprivation affects prosocial behavior will be explored in future research.

Inconsistent with the hypothesis of this study, our results indicated that expressive suppression and empathy do not chain-mediate the association between relative deprivation and prosocial behavior. Expressive suppression did not significantly predict empathy and prosocial behavior, which may be influenced by contextual or cultural factors. Some studies have suggested that expressive suppression can be an effective emotion regulation strategy in certain contexts (Guo et al., 2023). For example, expressive suppression has been found to reduce negative emotion arousal more quickly than cognitive reappraisal among Chinese participants, although it also requires more cognitive resources (Yuan et al., 2015). Therefore, it is unclear whether the use of expressive suppression strategies can effectively regulate personal distress in response to negative stimuli during the empathy process. Currently, empirical findings on the effects of expressive suppression strategies are inconsistent, indicating the need for further exploration of potential moderators in future research.

### 5.3. Research implications

Theoretically, the present study provides evidence on the association between relative deprivation and prosocial behavior in the adolescent populations. We explored the chain-mediating effects of two emotion regulation strategies on empathy and tested new mediating pathways that could reveal the intrinsic association between relative deprivation and prosocial behavior in adolescents. The results support the social comparison theory, the broaden-and-build theory of positive emotions, and integrative account of empathy and emotion regulation, providing new insights into the dynamics of prosocial behavior.

Practically, by understanding the effect of emotion regulation strategies and empathy on the association between relative deprivation and adolescent prosocial behavior, school psychologists can design intervention programs targeting the moral affect and prosocial behavior among secondary students. For example, a cognitive reappraisal mental health course could be implemented as an intervention for promoting prosocial behavior among adolescents, especially among those with high relative deprivation. In addition, cognitive reappraisal training can be provided as an adjunct to empathy training for adolescents.

### 5.4. Limitations

It should be noted that the present study has the following limitations. First, the results of this study were based on cross-sectional data, thus preventing the establishment of causal relationships between the variables. Therefore, future research should consider employing longitudinal designs for testing the hypothetical models. Second, the data pertaining to the variables were collected using subjective reporting methods. Therefore, objective data must be obtained by combining multiple methods such as parent, teacher, and peer evaluations to reduce the social praise effect. Additionally, the participants in the same school inevitably had consistent group characteristics, which may have affected the stability and generalizability of the present results. Whether the models hypothesized in this study holds true for a wider range of adolescents remains to be further tested. Finally, other variables that may influence the findings, such as the socioeconomic status of the study participants, were not collected in this study, and this aspect should be addressed in future studies.

## 6. Conclusion

In summary, the present study tested two chain-mediation models to explore the association between relative deprivation and adolescent prosocial behavior. Both cognitive reappraisal and empathy separately mediated the association between relative deprivation and adolescent prosocial behavior, whereas expressive suppression did not. Additionally, cognitive reappraisal and empathy chain-mediated the association between relative deprivation and adolescent prosocial behavior. Despite some limitations, the present study contributed to a better understanding of the association between relative deprivation and prosocial behavior, and the results emphasize the integrative role of empathy and emotion regulation as the underlying mechanism. This study also provides evidence that can form the basis for interventions that promote prosocial behavior among adolescents.

## Data availability statement

The datasets presented in this study can be found in online repositories. The names of the repository/repositories and accession number(s) can be found at: https://osf.io/bn9pm/files/osfstorage.

## Ethics statement

The studies involving humans were approved by the Ethics Committee of the School of Psychology, Fujian Normal University. The studies were conducted in accordance with the local legislation and institutional requirements. Written informed consent for participation in this study was provided by the participants’ legal guardians/next of kin.

## Author contributions

YX wrote the initial draft of this manuscript. SC and XS collected data for the study. DY supervised the project and provided critical revisions. All authors contributed to the article and approved the submitted version.
