# Corrigendum: Cognitive reappraisal and empathy chain-mediate the association between relative deprivation and prosocial behavior in adolescents

**DOI:** 10.3389/fpsyg.2024.1466931

**Published:** 2024-08-06

**Authors:** Yanfeng Xu, Sishi Chen, Xiaojie Su, Delin Yu

**Affiliations:** ^1^School of Psychology, Fujian Normal University, Fuzhou, Fujian, China; ^2^Normal College, Urumqi Vocational University, Urumqi, Xinjiang, China

**Keywords:** relative deprivation, cognitive reappraisal, expressive suppression, empathy, prosocial behavior

In the published article, there was an error in the Funding statement. The grant number stated for the “Education Reform Project of Psychology Teaching Steering Committee of Higher Education Ministry of Education (No. 2022012)” was incorrect. The correct Funding statement appears below.

